# Injury Prevention for Ski-Area Employees: A Physiological Assessment of Lift Operators, Instructors, and Patrollers

**DOI:** 10.1155/2013/121832

**Published:** 2013-07-25

**Authors:** Delia Roberts

**Affiliations:** School of University Arts and Sciences, Selkirk College, 301 Frank Beinder Way, Castlegar, BC, Canada V1N 4L3

## Abstract

*Background*. Momentary lapses in concentration contribute to workplace accidents. Given that blood glucose (BG) and hydration levels have been shown to affect vigilance, this study proposed to investigate these parameters and functional movement patterns of ski-resort workers and to determine whether an educational program to stabilize BG and hydration and encourage joint stability had an effect in decreasing occupational injuries. *Methods*. Seventy-five instructors, patrollers and, lift-operators at five snowsport resorts were evaluated for BG, vigilance, workload, dietary/hydration practices, and functional-movement patterns. Injury rates were tabulated before and after an educational program for nutrition and functional-movement awareness and compared to other resorts. *Results*. Workers showed poor stability at the lumbar spine, knee, and shoulder. BG levels were normal but variable (%CV = 14 ± 6). Diets were high in sugar and fat and low in water and many nutrients. Medical Aid and Lost Time claims declined significantly by 65.1 ± 20.0% (confidence interval −90.0% ≤*μ* ≤ −40.2%) in resorts that used the educational program whereas four control resorts not using the program experienced increases of 33.4 ± 42.9% (confidence interval −19.7% ≤*μ* ≤ −86.7%; *F*[2,12] = 21.35, *P* < 0.0001 ) over the same season. *Conclusion*. Provision of snowsport resort workers with educational programs encouraging hydration, diet to stabilize BG, and functional-movement awareness was effective in reducing worksite injuries in this population.

## 1. Background

Snowsport resort workers have one of the highest occupational injury rate classifications of all sectors. The average annual provincial claims cost over the five years preceding 2012 was $2.3 million in British Columbia alone, with an injury rate of 8.0 claims per 100 estimated person years of employment [1]. This is well in excess of the all industry rate of 2.3 claims per 100 estimated person years of employment. Furthermore, although the industry has implemented new safety programs, neither the injury rates nor costs have declined significantly, with 2012 having the second highest costs in the five-year time span [1]. 

Very little work has been done to examine the etiology of these injuries. Sixty percent of professional ski instructors reported a history of pain [2]. Similar results were seen in ski guides [3] who also showed low blood sugar levels, inadequate water intakes, and poor balance and proprioception of the knee and spine. Following a corrective educational program, injury rates in this group declined to less than half the original rate [3]. Similarly, a study involving ski instructors and patrollers indicated that increasing awareness of postures associated with a high risk of anterior cruciate ligament injury reduced the occurrence of these injuries by 62% [4]. Hodges and Richardson [5] have shown that while pain may resolve after injury, the timing delay in the protective feed-forward muscle activation may not be restored. It has been proposed that correcting these movement patterns and increasing positional awareness may, however, be important for preventing injuries of the lower limb [6].

Another potential contributing factor to occupational injuries is the impact of mild hypoglycemia [7–9] and dehydration [10] on measures of psychomotor vigilance including cognition, attention, and motor reaction time [11]. Falls to floor, walkway, or other surfaces or onto or against an object have been linked to momentary lapses of attention [12]; hypoglycemia [7–9, 13] and blood glucose variability have been shown to result in impairment of reaction time, cognition, memory, and decision making [14].

## 2. Objectives

Given both the prior injury histories and frequent chronic pain in this occupational sector as well as restricted access to food and fluids during the day, it may be that impaired proprioception and psychomotor vigilance are factors in the high injury rate seen in snowsport resort employees. In order to investigate the possible contribution of these parameters to injuries in this population, this study aimed to evaluate the regional relative risk of the spine and peripheral joints and the glycemic and hydration levels and coincident motor reaction time and vigilance levels in ski and snowboard instructors, patrollers, and lift attendants. The primary objective was to determine if injury rates are reduced following exposure to a program of corrective joint stability exercise and dietary counseling to stabilize blood glucose and hydration.

## 3. Methodology

### 3.1. Subjects

Five volunteers in each job designation (lift attendant, instructor, and patroller) were recruited at each of five snowsport resorts (*n* = 75) in the fall of 2010. The selection of participants was based on the work schedule for the testing dates and selection by each of the department supervisors. Informed consent was obtained and the rights and privileges of the volunteers safeguarded at all times. Independent ethical review was conducted by the Selkirk College Research Ethics Committee. The study design was a prospective cohort; during the first phase a descriptive assessment of a population sample was made; during the second phase a new industry-selected sample received the intervention. The outcome measures were the total number of injuries in all patrollers, instructors, and lift operators in a year-one to year-two comparison, as well as a cross-sectional comparison over the second season with resorts that did not utilize the program. The mean base and summit elevations of the 5 resorts were 1465 ± 279 m and 2305 ± 349 m, respectively. All subjects were well acclimatized at the time of on-snow testing.

### 3.2. Data Collection Season One

**Figure 1:**
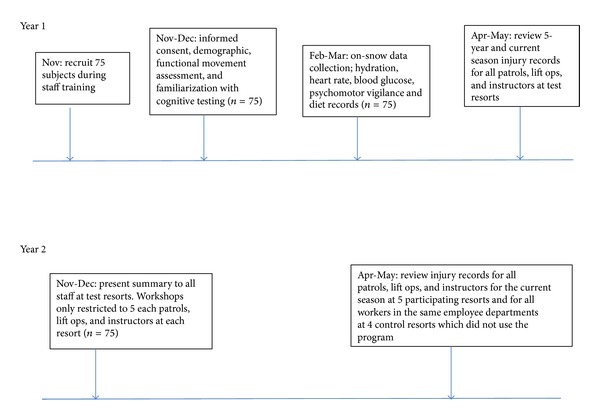
The experimental timeline.

The initial data collections occurred within the first month of the winter season at each resort (Figure 1) and were located in the base lodges. Demographic information including body composition estimated by bioelectric impedance (Omron Model HBF-306CAN, Scarborough, ON, Canada) and interviews for injury history and daily activity habits were collected. Learning trials for psychomotor vigilance testing were conducted and functional movement assessments were performed. On-snow data collections were carried out throughout the season (Figure 1). 

### 3.3. Functional Movement Assessment

Subjects completed a series of functional movements to evaluate the ability to actively maintain the test joint segment (knee, lumbar spine, or shoulder) in an anatomically neutral position while challenging it with independent movement at the adjacent joints, as would be required during normal daily work. These assessments were adapted from tests developed by Performance Stability (Ludlow, UK), which has previously been validated for use in functional movement assessment [15–19]. 

In brief, regional relative risk was assessed by evaluating the ability to control movement at the knees, lumbar spine, and shoulder during nonfatiguing slow movements, as well as with fatigue during rapid movements. Prevention of a defined motion at the site of the regional link was rated as a pass (able to demonstrate good active control) or fail (unable to actively prevent uncontrolled movement). All tests were filmed from two angles, lateral and either anterior or posterior view, and scored according to a predefined set of criteria by two independent assessors. The desired movement was demonstrated immediately prior to performing the test; the subject then attempted the movement with correction until being confident about the task. Verbal feedback was provided throughout the learning phase. 

The first test evaluated control of flexion and rotation at the lumbar spine during the first 50° of hip flexion. The subject stood with one foot positioned two foot lengths posterior to the lead foot while holding a 10 kg bar (as in lifting a pair of skis). The weight held in this position, anterior to the knee, increased flexion load on the lumbar spine. The posterior leg was held in the extended position and raised off the ground, thus increasing the rotational stress at the lumbar spine and the hip. The task was to squat three times to 45° of knee flexion on each side without rounding at the lumbar spine or rotating the pelvis. Inefficient control of hip rotation during this squat was of concern as it produces increased torsional stress on the knee joint, which was viewed as displacement of the knee from midline.

The second test was an alternating lunge split jump. This test assessed the ability of each subject to control knee, pelvis, and lumbar spine positioning during forces generated with jumping and eccentric loading during landing (as in downhill skiing or boarding). The subject started with a distance equal to the measure of three of their foot lengths between the front and back feet and performed a series of 10 alternating split squat jumps, landing with the anterior knee at 90° and immediately lifting the heel of the front foot. This landing position was held for five seconds prior to the next jump, where the lead foot was switched while in the air. The destabilized hold position (with the heel raised) for each of the 10 jumps without rest increased the time under tension and induced fatigue and also tested for the ability to limit rotational stress from the distal joint complex on the knee (as would occur during turning on skis or a snowboard or during destabilization due to a slip or trip). Other movements evaluated were whether the lumbar spine flexed, extended, and/or laterally bent during jumping or landing and if the hip/leg musculature was able to keep the front knee in midline throughout the test. This was of interest because hip stability has been correlated with injuries to the anterior cruciate ligament of the knee [20].

The final test evaluated the ability to maintain scapular-thoracic stability and control of lumbar extension with loaded overhead arm actions. Subjects began in a bridge position from knees and elbows with the lumbar spine in a neutral posture. One arm was raised overhead for 3 successive repetitions prior to switching sides. Raising the arm increased extension and rotational forces on the lumbar spine. With poor stability the scapula(e) of the stabilizing arm and/or the raised arm were/was observed to lose contact with the thoracic wall, resulting in increased muscular stress on the cervical spine and poor biomechanical patterning at the glenohumeral complex.

### 3.4. Blood Collection and Analysis

Capillary blood samples were collected from fingertip using standard sterile procedure. Samples were analyzed immediately using the Contour Glucose Monitoring System (Bayer Healthcare LLC, Toronto, ON, Canada) calibrated twice daily. Eight-hour fasting blood samples were collected at the start of the day; subjects were then encouraged to follow their usual dietary and hydration practices. Approximately every 2 hours thereafter the research team visited each participant wherever they were working to collect a blood glucose sample and complete the psychomotor vigilance testing.

### 3.5. Psychomotor Vigilance Testing and Symptom History

Psychomotor vigilance testing was conducted using Brain Checkers software, Version 3.01 (Behavioral Neuroscience Systems LLC, Springfield, MO, USA) run on Palm Tungsten E2, (Palm Inc. Milpitas, CA, USA). The tests were designed to evaluate simple reaction time, 2-choice reaction time, short-term memory, and visual information processing. The first test was 30 sec in duration and measured simple reaction time as speed of motor response to a repeated unexpected visual stimulus. The second test assessed the ability to recall and process numerical information accurately in a 2-minute continuously running 2-choice reaction time test. These same parameters were further evaluated during the third test, which relied on visual pattern recognition. Reported parameters include the mean reaction time for each session and parameter, as well as a calculated Tput statistic that represents the number of correct responses per minute of available time to respond. The complete protocol has been previously described [14] and validated elsewhere [21, 22].

Each subject completed a familiarization session at the start of the season during the initial data collection and functional movement assessment, consisting of five trials of each of the three tests. Previous work has shown that after 3 trials the learning curve levels off, and after five trials there is no further improvement [14, 22]. During the on-snow data collections, one trial of each test was completed every two hours, as described in the blood collection section above. 

 Participants were also asked to record and/or report symptoms of falling blood glucose and counterregulatory hormones or dehydration such as hunger, fatigue, irritability, inability to focus, dizziness, confusion, nausea, tremor, and sweating at each on-snow testing session.

### 3.6. Hydration

Body mass was recorded prior to the start of the workday and again just prior to the worker leaving for home (bladder empty, subject wearing only underwear) using the SR Model SR241 scales (accuracy = 0.2% ±1 digit, resolution = 0.1 kg, SR Instruments, NJ, USA). Hydration level was estimated based upon the assumption that each kilogram loss represented a reduction in body water by 1 liter [23]. 

### 3.7. Dietary Analysis

 Two- or 3-day diet records were completed by each participant according to specific criteria for accuracy; careful instructions on how to measure, define, and record all food and drink consumed were given in both written and oral formats. Verbal 2-hour diet recall was also taken at the time of each blood glucose sample. Dietary analyses were made on the basis of a minimum of three full days using Diet Analysis Plus, Canadian version 9.0 (Wadsworth/Thompson Learning, Scarborough, ON, Canada). The nutritive requirements were based upon the Dietary Reference Intakes (DRI) [24]. 

### 3.8. Workload Assessment

Workload was assessed by heart rate, recorded every 15 seconds for two full workdays on each participant (Actiheart system, Mini Mitter Co. Inc, Bend, OR, USA). 

### 3.9. Injury Monitoring

Five years of historical injury records were obtained from each of the five test resorts (2005/06–2009/10 winter seasons). Only records for patrollers, lift operators, and snowsport instructors were examined. Claims for all workers in these three job classifications were categorized based upon First Aid (FA) only (report made but no followup required), Medical Aid (MA) when the injury was examined by a physician (may have resulted in diagnostic tests, rehabilitative treatment, and/or modified duties), or Lost Time (LT) where the worker was unable to work and a claim for lost wages was made. The number of claims in each category was recorded for each year and a 5-year average was calculated. This process was repeated at the end of the 2010/11 season for claims made during the first year of the study and again at the end of the 2011/12 season for claims made during the season in which the preventative program was administered. 

In addition, a request was sent out through the industry safety association for resorts that had not participated in the study to share their injury data for comparative purposes. Four resorts submitted summary data listing the number of injuries in each category for all patrollers, instructors, and lift operators working at each of the resorts during the two seasons over which the study was conducted. There was no limitation on these areas implementing their own safety programs. Since the areas ranged in size, number of employees, and employee work exposure and it was not possible to establish whether the same criteria for classification of injuries were used at all areas, the comparisons between injury rates were made as relative change over time within each resort.

### 3.10. Season Two

A worksite health and wellness program designed to build healthy eating and drinking behaviors within the lifestyle and resources of a young snowsport resort worker and to establish neutral postures and stability at the lumbar spine, knee, and shoulder was offered to all employees at the five test resorts. During fall staff training, a short presentation (30 minutes–1 hour) open to all staff described the findings of the first year of the study, and briefly described the nutrition and proprioceptive program. In addition, each of the five participating snowsport areas selected 15 new volunteers (five in each job category of lift operators, patrollers, and instructors) to receive printed materials and attend half-hour workshops on nutrition and proprioception.

The nutrition workshop covered the three basic types of foodstuffs (carbohydrates, proteins, and fats) and their digestion times. The main points of focus were on how to maintain euhydration and to consume small low-fat, complex carbohydrate, and lean protein snacks every 2-3 hours during the workday to stabilize blood sugar. Glycogen replenishment was also recommended following high-activity days. Strategies for shopping, cooking, and packing of healthy meals on a budget and while living at a snowsport resort were also provided. Discussion on limiting alcohol and other recreational substance use was encouraged.

The proprioception workshop focused on learning how to position one's body into a posture with the spine and pelvis in neutral position. Participants then learned to stabilize this neutral posture while standing (in ski or snowboard boots), sitting (as on a chairlift), or moving (while performing work tasks, carrying a pack, skiing, or riding). Simple proprioceptive drills to help increase stability at the lumbar spine, hip, knee, and shoulder were presented and participants were encouraged to utilize the drills regularly. The increase in functional movement stability was stressed from the perspective of enhanced performance as well as a decrease in the risk of injury.

Although only 15 workers at each resort attended the workshops and were provided with the printed materials, the workshop participants were encouraged to share the information within their departments. Supervisors from each outdoor department were also invited to share in the more detailed information and were provided with strategies to support the program on a biweekly basis throughout the season. In addition, all workers had access to a Facebook page for the program with regular postings on tips, recipes, exercises, fun activities, and other engaging platforms to support the program focal points of quality nutrition, hydration, fitness and proprioception, and a healthy lifestyle.

### 3.11. Statistical Analysis

All means were reported as mean ± 1 SD. After testing for normalcy and equal variances, injury rate comparisons were made using analysis of variance (ANOVA), followed by paired (historical) or independent (between areas) *t*-tests with the Bonferroni correction for significant differences. The relationship between the mean variability in blood glucose levels (reported as % coefficient of variation (CV) = [standard deviation/mean]∗100%) and the psychomotor reaction time measures was explored using a 2-way analysis of variance (regression analysis). All tests were run using Minitab 16.2.2 (State College, PA, USA), with the level of significance set at *P* ≤ 0.05. 

## 4. Results

**Figure 2:**
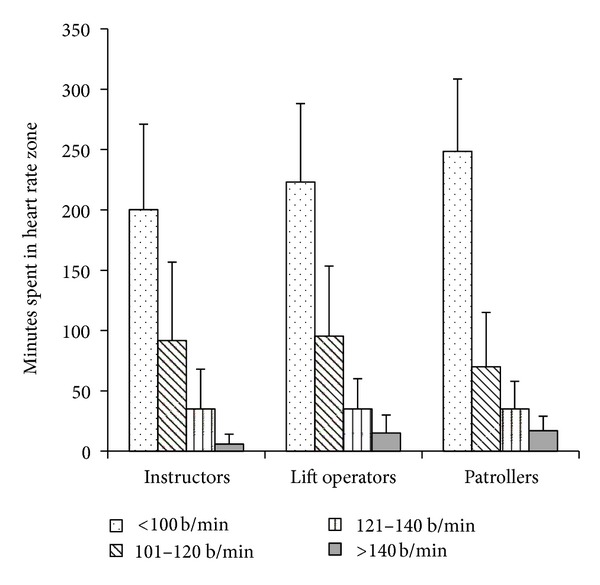
Mean ± SD time spent (minutes) in each heart rate zone for each of the three occupational groups for a day of work. b/min: beats per minute. Data collected at five snowsport resorts in Western Canada during the first season of the study (*n* = 75).

Demographic information is presented in Table 1 (data from year 1); the majority of participants were between the ages of 20 and 40 years (range 18–76 years), nonsmokers, and, except for instructors, predominantly males. Instructors also tended to have the most experience; only one individual was in their first year of teaching, while 15/25 lift operators had one or less years of experience (range 0–55 years for patrollers, 0–16 years for instructors, and 0–4 years for lift operators). Heart rates during the workday are presented in Figure 2 (data from year 1); the majority of the day was spent at heart rates that were below 120 b/min with a relatively short exposure to more intense levels of exercise. There were no statistical differences between the three groups; however, based upon daily cumulative time spent above a heart rate of 120 b/min, 61% of patrollers met the American College of Sports Medicine-recommended 30 minutes of moderately vigorous physical activity (3–5.9 METs) daily for good health [25], whereas only 43% and 45% of instructors and lift operators, respectively, met this criteria while at work. Less than half of lift operators and instructors reported participating in any physical activity outside of work, while 72% of patrollers were active recreationally (Table 1). The activity levels at work and recreationally corresponded to body composition findings, with only 13% of patrollers being overweight, while 41 and 42% of instructors and lift operators respectively had estimated body fat levels that placed them into the overweight category (Table 1). A very high proportion of participants reported having a history of injuries of the lower back, knee, or shoulder, as well as chronic pain at one or more of these joints (Table 2—data from year 1). Patrollers reported injuries of the shoulder and back most frequently, instructors reported injuries of the knee as the most prevalent, and lift operators reported the highest frequency of injuries to be in the knee and back. Only 3/75 individuals tested were able to perform at least one of the performance matrix tests and maintain stability at all joints tested (shoulder, hip, knee, and lower back) (Table 3—data from year 1).

**Figure 3:**
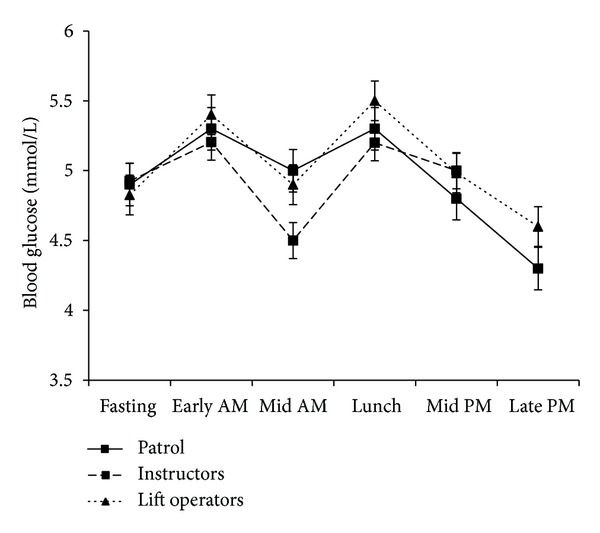
Mean ± SD blood glucose levels during a day of work for patrollers, instructors, and lift operators while consuming their normal diet. Data collected at five snowsport resorts in Western Canada during the first season of the study (*n* = 75).

All of the study participants in year one had normal blood glucose levels; the mean ± SD fasting value was 5.0 ± 0.6 mmol/L; levels during the workday are presented in Figure 3 (data from year 1). The mean %CV ± SD over the day was 15 ± 6% (range 3.2–7.3 mmol/L) for patrollers, 13 ± 7% (range 3.9–8.7 mmol/L) for instructors, and 15 ± 6% (range 2.6–7.2 mmol/L) for lift operators. Excluding the first glucose sample of the day to account for the fasting condition had very little impact on the results; therefore all samples were included in the statistical analysis. Nineteen percent of employees demonstrated at least one sample/day where blood glucose levels were in the hypoglycemic range [26] and 51% of employees reported two or more symptoms in conjunction with blood sugar levels that were below their fasting level.

**Figure 4:**
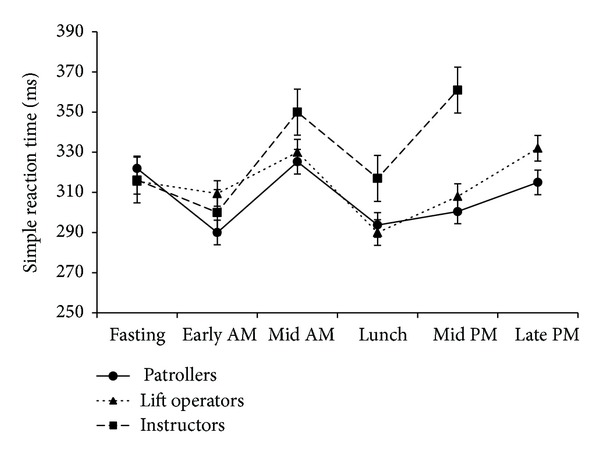
Mean ± SD simple reaction time responses (msec) during a day of work for patrollers, instructors, and lift operators. Data collected at five snowsport resorts in Western Canada during the first season of the study (*n* = 75).

**Figure 5:**
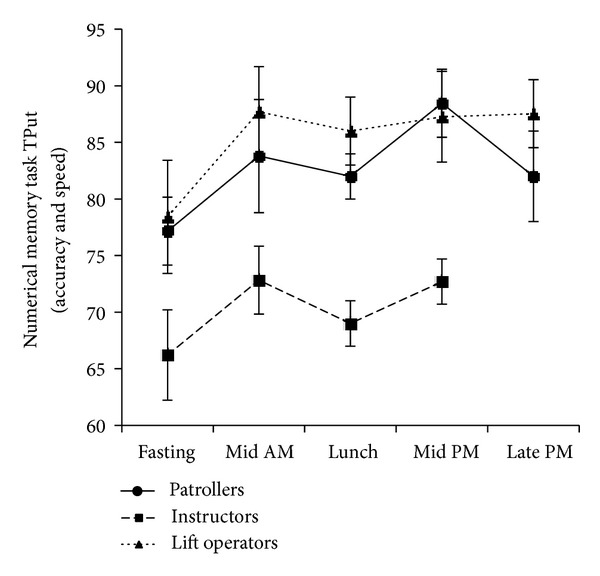
Mean ± SD two-choice reaction time in response to a numerical memory task, normalized for accuracy and represented as throughput (TPut), a unitless parameter. Data collected at five snowsport resorts in Western Canada during the first season of the study (*n* = 75).

**Figure 6:**
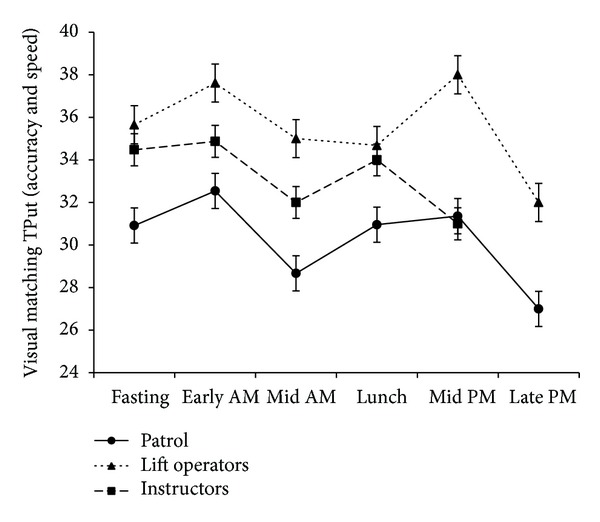
Mean ± SD two-choice reaction time in response to an unexpected complex visual pattern normalized for accuracy and represented as throughput (TPut), a unitless parameter. Data collected at five snowsport resorts in Western Canada during the first season of the study (*n* = 75).

The results of the reaction time and cognitive processing testing are presented in Figures 4–6 (data from year 1). Simple reaction time is given in milliseconds (Figure 4), with a lower score indicating a faster reaction time. The results of the two complex reaction time tests are presented as normalized for accuracy of response with a higher score indicating a faster and more accurate response.  Figure 5 shows the results for the numerical memory task, Figure 6 the visual pattern recognition task. Although Figures 3–6 follow similar patterns, no statistically significant correlations were found between psychomotor vigilance scores and blood glucose levels.

**Figure 7:**
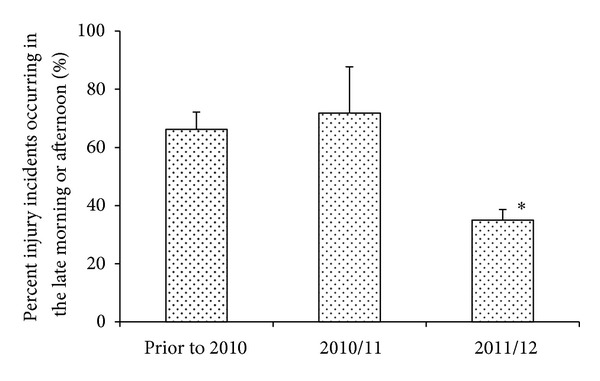
The mean ± SD number of injury incidents occurring at a time of day when workers were likely to have been hypoglycemic (10:30–12:00 and 15:30–17:00) as a percentage of the total number of injury incidents reported for that year. *Significantly less than previous years, *F*[2,12] = 19.50, *P* < 0.0002. Data for all patrollers, lift operators, and instructors working at five snowsport resorts in Western Canada.

A historical examination for time of day of injury was also made for the five participating resorts. Over a 6-year time span (2005–2011), 69 ± 12% of injuries occurred during a total of three hours, composed of late morning or afternoon (Figure 7), when blood sugar levels may be expected to be at their lowest (confidence interval 61% ≤ *μ* ≤ 82%). In contrast, only 33 ± 2% of injuries occurred within this same time frame during the 2011/12 season (confidence interval 22% ≤ *μ* ≤ 44%; *F*[2,12] = 19.50, *P* < 0.0002).

A summary of the diet records is presented in Table 4 (data from year 1) along with the DRI for comparison. In general, the reported diets were high in total fat, saturated fat, cholesterol, and sugar; the mean daily intake exceeded the DRI by 29% ± 9% (total fat), 50% ± 15% (saturated fats), 58% ± 20% (cholesterol), and 26% ± 19% (total sugar), respectively. In contrast, the daily intake of fruits and vegetables was lower than that recommended by the Canadian Food Guide leading to suboptimal intakes of a number of important nutrients including total fiber, vitamins A, D, and E, magnesium, and potassium. In addition, female lift operators and instructors had inadequate intakes of folate, calcium, and iron. The mean reported intake of water was 75% ± 20% of the DRI, which was reflected in the mean body mass losses from morning to evening in all three groups (patrollers −2.0% ± 1.9%, instructors −1.9% ± 2.0%, and lift operators −2.0% ± 3.0% body mass, resp.). Although the mean value estimates only mild dehydration, 20% of individual patrollers, 26% of instructors, and 35% of lift operators lost more than 2% of their body mass over the workday.

**Figure 8:**
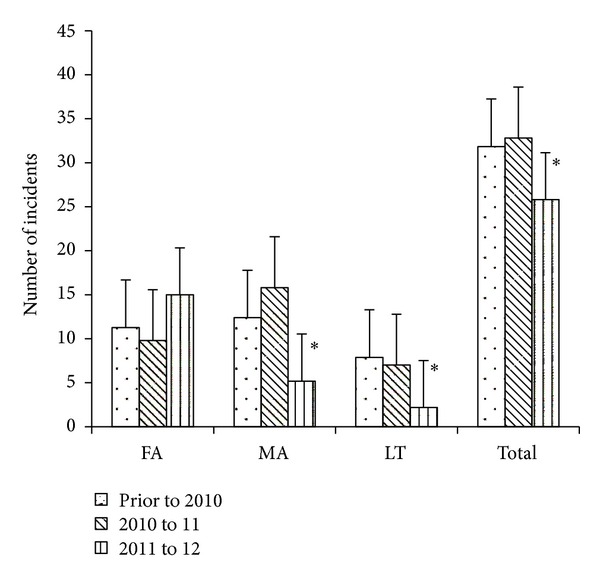
The mean ± SD number of injury incidents for all patrollers, lift operators, and instructors working at five test snowsport resorts prior to and following implementation of the dietary and proprioceptive program (2011/12 season). The prior to 2010 data include up to five years of historical records for all workers in the three job categories. FA = First Aid report only, MA = Medical claim only, and LT = Lost time. *Significantly less than previous seasons, where *P* < 0.04 for the reduction in MA, *P* < 0.006 for LT, and *P* < 0.04 for the reduction in total claims.

Highly significant reductions in medical aid and lost time claims were observed for all employees in the three test job classifications of patroller, lift operator, and instructor, at all five resorts where the nutrition and proprioceptive program was provided (Figure 8). Two resorts achieved zero Lost Time claims in these departments for the season. Overall, recordable incidents in these three job classifications were reduced by 65.1% ± 20.0% from the 2010/11 season compared to the 2011/12 season (confidence interval −90.0% ≤ *μ* ≤ −40.2%) compared to an increase of 33.4% ± 42.9% (confidence interval −19.7% ≤ *μ* ≤ 86.7%) over the same time frame in resorts which did not use the program (*F*[2,12] = 21.35, *P* < 0.0001). There were no significant differences within the three job classifications (patrollers, lift operators and, instructors) for the distribution of injury claims between FA, MA, and LT or in the degree of reduction from the 2010/11 season compared to the 2011/12 season (data not shown).

## 5. Discussion

The primary outcome of this investigation was a large reduction in the number of recordable incidents in patrollers, instructors, and lift operators working at five snowsport resorts in Western Canada during a season where a nutritional and proprioceptive training program was used (Figure 8). This observation is strengthened by the overall increase in claims for the three test job classifications in four other snowsport resorts located in the same geographical region, that did not use the educational program, over the same time frame.

These findings support previous reports that stabilizing blood glucose levels can significantly impact measures of vigilance including reaction time, cognition, memory, and decision making [3, 7–11, 14, 27–29]. Although the number of incidences of true hypoglycemia (blood glucose levels in the 3.3–3.8 mmol/L range [26]) with concomitant self-reported symptoms were below 20% in the first season of the current study, previous work has shown that decreasing the variability of blood glucose from 18% to 12% had a significant effect in improving all measures of psychomotor vigilance [14]. In the current study the 14 ± 6% CV in blood glucose levels and the 2-3 hour spot-sampling protocol did not allow us to establish a correlation between glycemic level and our measures of vigilance. However, in studies with physicians [14] or truck drivers [28] where we compared the results of a day of self-selected diet with a day of imposed feeding, the stabilization of blood glucose did correlate with an improvement in scores on every parameter measured including reaction time, memory, accuracy, and decision making of a magnitude equivalent to the norms for two age group levels [14]. Given that the dietary records of the ski resort employees tested during the first season indicated that overall, the consumptions of saturated fats and sugars were well in excess of the DRI, it is not surprising that a culturally specific educational program that provided quality information on the performance benefits of stabilizing blood sugar by consuming small frequent meals composed of complex carbohydrates and lean proteins, fresh fruits, and vegetables would have a beneficial effect. The primary outcome measure of reduced injuries in the ski areas that provided nutritional training (whether the comparison was made to a five-year average, the previous season, the timing of injuries to late morning or afternoon, or to other ski resorts over the same season) demonstrates a strong argument for the role of blood glucose variability in the etiology of workplace injuries and accidents in this population. 

The possibility that some of the improvements in psychomotor vigilance in the previous studies and the reduction in injury rates observed in the current study were due to maintaining euhydration cannot be excluded. Based on the use of change in body mass from the start of the day to the end of the workday, the estimated mean level of dehydration in the first season of the current study was close to the 2% loss at which dehydration has been shown to impair cognition and reaction time [10, 11]; approximately 27% of participants exceeded this level. The dietary recommendations utilized in the educational program delivered at the start of the second season emphasized the need for ski resort workers to carry water with them during the day and to consume small amounts on a frequent basis. In the studies with physicians [14] and truck drivers [28] the enforced feeding regime included 250–300 mL water/hour. This level of intake was sufficient to maintain euhydration, and both of these studies showed enhanced psychomotor vigilance with restoring hydration as part of the treatment. 

 In addition to stabilizing blood sugar and maintaining hydration levels, the very positive outcome of reduced injuries in patrollers, lift operators, and instructors working at the five test resorts using the training program in the second season was likely due to the inclusion of proprioceptive exercise that was specific to the movement challenges faced by these workers, but which were simple to use and could be incorporated into the workday. Although (as for the nutritional training) we have no direct evidence of altered behavior in those undergoing the training, given the frequency of reported previous injuries (Table 2) along with the poor performance at the functional movement tests (Table 3), it would seem likely that correcting dysfunctional movement patterns may have been a contributing factor to the reduction in injury rates in the five test ski resorts. 

 Several groups have shown that individuals with a previous history of low back pain who are asymptomatic may have the same amount of total hip and lumbar range of motion available during forward bend as healthy subjects, but they moved differently in the first 40° of forward flexion the noninjured group, producing increased flexion stress on their lumbar spine in the early part of a functional bend [30, 31]. Altered movement strategies can also have an effect on peripheral joints. There is evidence associating abnormal scapular positions and motions with a variety of shoulder pathologies [32, 33] and poor stabilization of the hip with increased potential for serious injury at the knee [20, 34]. The supposition that correcting these altered movement patterns can help reduce subsequent injuries is supported by a large body of evidence linking the positive benefits of regular proprioceptive exercises in reducing sport injuries [20, 35–38]. 

 Many of these studies have looked at using balance, plyometric, and agility components to reestablish recruitment patterns and improve joint stability following soft tissue injury [35, 37, 38]. Proprioceptive receptors in muscle, tendon, and joint capsules are responsible for coordinating muscular contraction so that a joint is stabilized as applied forces increase [39]. In a healthy system muscle will contract within 10 msec of the beginning of an applied force, well within the approximately 50 msec required for the forces to reach peak levels [36, 40]. Unfortunately these small proprioceptive nerve endings are easily damaged, resulting in a slowing of the stabilization response or even contraction of the wrong muscles [41]. Fatigue [42, 43], trauma, edema, and bruising [44] will all reduce the speed of response such that the pattern of muscle recruitment is slowed and less likely result in stabilization of a joint prior to the development of peak force, leaving the joint susceptible to injury. 

 Fortunately, research has also shown that proprioceptive training can increase the speed of recruitment, restoring the protective reflex [36]. On the basis of these findings, it would seem that the scarcity of application of proprioceptive drills combined with education and training of movement strategies in occupational injury prevention programs is unfortunate, as these techniques are likely to have a pronounced effect on improving the performance of daily work tasks and reducing the likelihood of injury. The findings of the current study suggest that this would seem to be an area worthy of further investigation. 

As with most applied research, there are some methodological considerations regarding the findings of the current study. Due to the nature of the worksite environment and the culture of the subject group there were many uncontrolled variables including but not limited to weather, exposure to moderate altitude, snow conditions, equipment, work hours, extracurricular activities, and a seasonal work force. Employee turnover prevented retesting of subjects or followup to determine the effect of employee training on behavior. In addition, each resort had its own procedure for classifying and recording injury events, making it difficult to compare results between areas. These factors notwithstanding, the objective observations in the significant reduction in the number of injuries sustained in all five resorts that utilized the nutritional and movement training program speak strongly in favor of the positive effect of a culturally and contextually specific health and wellness program in reducing injuries in patrollers, instructors, and lift operators working at snowsport resorts. The study findings indicate that providing these young workers with quality information in a format that is meaningful to them was effective in decreasing occupational injuries in spite of the inability to ensure that all employees complied or were even exposed to the training in an ideal manner. 

The integration of occupational injury prevention and worksite health promotion is becoming an area of focus, as medical and insurance costs continue to rise. The components of the program utilized in the current study are in compliance with the recommendations for an integrated worker health program as outlined by CDC NIOSH's Total Worker Health Program [45], and the study outcomes provide further support for the effectiveness of worksite-based injury prevention programs that integrate health- and wellness-based lifestyle education with injury prevention.

Given the high rates of injury common at snowsport resorts and the escalating costs of these injuries, combined with limited recourses available for employee training and the fact that compliance with healthy lifestyle behavioral change programs is often poor, the inclusion of this type of low-cost, highly effective, workplace intervention is highly recommended.

## 6. Conclusion

 The findings of the current investigation provide evidence that integrated worksite health and wellness programs that encourage healthy eating patterns to stabilize blood glucose levels and maintain euhydration, combined with increased postural awareness and joint stability exercise, can help to significantly reduce occupational injury rates in ski resort workers. The difficulties of conducting research in a workplace setting notwithstanding, further investigations into these types of injury prevention programs are warranted.

## Figures and Tables

**Table 1:** Demographic information for the 75 study participants collected during the first season at five snowsport resorts in Western Canada. Data are presented as mean ± SD or as a percentage of the number of volunteers.

	Patrollers	Instructors	Lift operators
Age	30 ± 12 yrs	36 ± 16 yrs	23 ± 4.8 yrs
% Males	84%	54 %	78%
% Smokers			
Tobacco	0%	13%	17%
Other	28%	8%	40%
% Skiers	80%	56%	43%
% Telemark	20%	0%	0%
% Snowboard	0%	39%	47%
% Both board and ski	0%	4%	13%
Years of experience	2 ± 12 yrs	6 ± 4 yrs	1 ± 1 yrs
Participates in recreational physical activity	72%	46%	33%
% Body fat			
Males	14.6 ± 5.4%	20.1 ± 6.7%	15.1 ± 6.7%
Females	16.6 ± 3.4%	24.4 ± 7.2%	27.3 ± 9.9%
BMI			
Males	23.9 ± 2.5	26.1 ± 2.6	24.9 ± 5.1
Females	21.7 ± 1.7	23.3 ± 5.4	25.5 ± 5.8

**Table 2:** Prevalence of patrollers, instructors, and lift operators reporting at least one previous injury reported as a percentage of those interviewed (*n* = 75). Data collected at five snowsport resorts in Western Canada during the first season of the study.

	Patrollers	Instructors	Lift operators
Previously injured	80%	92%	88%
Shoulder	45%	38%	37%
Back	45%	42%	42%
Knee	30%	50%	42%
Ankle	20%	42%	37%
Chronic pain	76%	92%	84%

**Table 3:** Functional movement analysis results for 75 snowsport resort employees. Data collected during the first season at five resorts in Western Canada. Scores are a unitless number representing the number of movement criteria displayed during the test. A higher score indicates a lower ability to stabilize the lower back, hip, knee, or shoulder joints during loaded movement.

Performance matrix	Patrollers	Instructors	Lift operators	% Participants* with good stability
Single leg knee bend (Score out of 7)	3.6 ± 1.7	4.3 ± 1.8	4.5 ± 1.5	7%
Lunge jump (Score out of 8)	3.8 ± 1.7	3.5 ± 1.5	4.9 ± 1.4	3%
Push-up arm lift (Score out of 7)	3.0 ± 1.4	3.2 ± 1.1	3.3 ± 1.5	11%

*The percentage of individuals who scored ≤1.0 on the Performance Matrix test.

**Table 4:** Dietary intake as reported from two- or three-day diet records for 75 patrollers, instructors, and lift operators at five ski resorts in Western Canada during the first year of the study. Data is represented as mean ± SD. The Recommended Dietary Allowance (RDA) is provided for comparison [24].

	Patrollers		Instructors		Lift ops		RDA	
	Men	Women	Men	Women	Men	Women	Men	Women
Kcal	3140 ± 762	2831 ± 525	2621 ± 726^∧^	1997 ± 486^∧^	2989 ± 762	1979 ± 70^∧^	2700 kcal	2100 kcal
Protein (g)	121 ± 26	113 ± 8	96 ± 17	75 ± 19	114 ± 33	64 ± 23	0.8 g/kg body wt	
Carbohydrate (g)	413 ± 129	360 ± 62	339 ± 127	256 ± 71	363 ± 93	271 ± 49^∧^	>130 g	
Total fat (g)	115 ± 40*	100 ± 27*	97 ± 31*	70 ± 18*	117 ± 41*	63 ± 16*	<25–35% calories	
Saturated fat (g)	39 ± 20*	30 ± 8*	31 ± 11*	21 ± 7*	39 ± 19*	22 ± 5*	<7% caloric intake	
Monounsaturated fat (g)	30 ± 16^∧^	29 ± 9^∧^	27 ± 13^∧^	21 ± 11^∧^	38 ± 16	19 ± 7^∧^	<10–25% calories	
Polyunsaturated fat (g)	18 ± 10^∧^	13 ± 4^∧^	19 ± 10^∧^	17 ± 10^∧^	22 ± 9^∧^	13 ± 2^∧^	<8–10% calories	
Trans fatty acid	0.8 ± 1.3	0.5 ± 0.8	0.4 ± 1.3	0.3 ± 0.4	0.9 ± 1.5	0.3 ± 0.4	<1% caloric intake	
Omega-6 linoleic acid (g)	14 ± 9^∧^	11 ± 4^∧^	12 ± 7^∧^	10 ± 6^∧^	16 ± 8^∧^	8 ± 7^∧^	17 g	12 g
Omega-3 linolenic acid (g)	1.2 ± 1.3^∧^	0.8 ± 0.6^∧^	1.2 ± 0.6^∧^	3.2 ± 6.8	1.3 ± 0.8^∧^	0.6 ± 0.4^∧^	>1.6 g	>1.1 g
Cholesterol (mg)	360 ± 171*	329 ± 101*	274 ± 156*	220 ± 170*	400 ± 228*	204 ± 141*	<200–300 mg	
Total fiber (g)	40 ± 18	38 ± 9	28 ± 7^∧^	24 ± 11^∧^	34 ± 15^∧^	19 ± 8^∧^	>38 g	>25 g
Total sugar (g)	156 ± 74*	142 ± 53*	157 ± 68*	99 ± 34	160 ± 45*	152 ± 45*	<25% caloric intake	
Total water (L)	1.5 ± 2.0^∧^	1.8 ± 1.2^∧^	2.0 ± 1.0^∧^	1.6 ± 0.6^∧^	2.2 ± 1.2^∧^	1.3 ± 0.1^∧^	3.7 L	2.7 L
Thiamin (mg)	2.0 ± 1.1	2.1 ± 1.6	1.8 ± 0.6	1.2 ± 0.3	1.8 ± 0.6	2.1 ± 0.8	1.2 g	1.1 g
Riboflavin (mg)	2.3 ± 1.2	3.8 ± 3.0	2.1 ± 0.5	1.4 ± 0.4	2.3 ± 0.7	2.3 ± 1.0	1.3 mg	1.1 mg
Niacin (mg)	28 ± 10	34 ± 9	24 ± 8	19 ± 5	27 ± 9	35 ± 26	16 mg	14 mg
Vitamin B6 (mg)	2.2 ± 1.1	2.4 ± 1.8	2.5 ± 2.6	1.6 ± 0.5	2.2 ± 0.7	2.4 ± 1.8	1.3 mg	
Vitamin B12 (mcg)	5.1 ± 2.8	6.9 ± 3.9	6.4 ± 6.3	2.6 ± 1.5	4.8 ± 2.8	3.9 ± 4.6	2.4 mcg	
Folate (DFE) (mcg)	456 ± 246	675 ± 337	441 ± 239	374 ± 161^∧^	566 ± 207	396 ± 213^∧^	400 mcg	
Vitamin C (mg)	155 ± 88	205 ± 96	169 ± 266	135 ± 119	153 ± 110	128 ± 28	90 mg	65 mg
Vitamin D (IU)	205 ± 226^∧^	257 ± 123^∧^	176 ± 265^∧^	112 ± 132^∧^	126 ± 165^∧^	143 ± 129^∧^	600 to 4000 IU	
Vitamin A (RAE) (mcg)	693 ± 576^∧^	803 ± 639	704 ± 334^∧^	722 ± 502	868 ± 483^∧^	286 ± 54^∧^	900 mcg RAE	700 mcg RAE
Alpha-tocopherol (Vit E) (mg)	7.4 ± 6.9^∧^	9.5 ± 4.8^∧^	5.1 ± 3.2^∧^	4.9 ± 3.6^∧^	9.8 ± 4.7^∧^	4.5 ± 3.3^∧^	15 mg	
Calcium (mg)	1209 ± 413	1286 ± 167	987 ± 506^∧^	876 ± 214^∧^	1079 ± 492	576 ± 149^∧^	1000 mg	
Iron (mg)	23 ± 7	23 ± 5	21 ± 7	16 ± 6^∧^	20 ± 5	14 ± 1^∧^	8 mg	18 mg
Magnesium (mg)	468 ± 215	438 ± 42	375 ± 124^∧^	288 ± 107^∧^	444 ± 144	262 ± 17^∧^	420 mg	320 mg
Potassium (mg)	4215 ± 1654^∧^	3882 ± 906^∧^	3271 ± 708^∧^	2438 ± 495^∧^	3771 ± 1100^∧^	3074 ± 241^∧^	>4700 mg	
Zinc (mg)	15 ± 7	14 ± 11	13 ± 3	8 ± 2	15 ± 5	9 ± 6	11 mg	8 mg
Sodium (mg)	3933 ± 1324*	3314 ± 1023*	2803 ± 1056*	3310 ± 1522*	3504 ± 1459*	3400 ± 1217*	<2300 mg	

*Exceeds the RDA for good health, ^∧^does not meet the lower limit of RDA for good health.
